# Improvement of care for the physical health of patients with severe mental illness: a qualitative study assessing the view of patients and families

**DOI:** 10.1186/1472-6963-13-426

**Published:** 2013-10-21

**Authors:** Fenneke M van Hasselt, Marian JT Oud, Anton JM Loonen

**Affiliations:** 1 Pharmacotherapy and Pharmaceutical Care, University of Groningen, Antonius Deusinglaan 1, Groningen 9713 AV, The Netherlands; 2 GGZ WNB, Mental health hospital, Postbus 371, Bergen op Zoom 4600 AJ, the Netherlands; 3 Department of General Practice, University Medical Center Groningen, Antonius Deusinglaan 1, Groningen 9713 AV, The Netherlands; 4 Delta chair on pharmacotherapy in psychiatric patients, Delta, Mental Health Hospital, Postbus 800, Poortugaal 3170 DZ, The Netherlands

**Keywords:** Community mental Health services, General practice, Health promotion, Patient participation, Qualitative research

## Abstract

**Background:**

Patients with severe mental illness (SMI) experience more physical comorbidity than the general population. Multiple factors, including inadequate seeking of healthcare and health care related factors such as lack of collaboration, underlie this undesirable situation. To improve this situation, the logistics of physical health care for patients with SMI need to be changed. We asked both patients and their families about their views on the current organization of care, and how this care could be improved.

**Methods:**

Group and individual interviews were conducted with patients and family of patients to explore their needs and preferences concerning the care for the physical health of patients with SMI, and to explore the shortcomings they had experienced. Using thematic analysis, responses were firstly divided into common topics, after which these topics were grouped into themes.

**Results:**

Three major themes for the improvement of the physical care of patients with SMI were found. Firstly, the reduced ability of patients with SMI to survey their own physical health interests requires health care that is tailored to these needs. Secondly, the lack of collaboration amongst mental health care professionals and general practitioners (GPs) hinders optimal care. Thirdly, concerns were expressed regarding the implementation of monitoring and supporting a healthy lifestyle. Patients with SMI welcome this implementation, but the logistics of providing this care can be improved.

**Conclusions:**

An optimal approach for caring for the physical health of patients with SMI requires a professional approach, which is different to the routine care provided to the general public. This approach can and should be accomplished within the usual organizational structure. However, this requires tailoring of the health care to the needs of patients with SMI, as well as structural collaboration between mental health care professionals and GPs.

## Background

Patients with severe mental illness (SMI) have more physical comorbidity than the general population [1]. This is caused by multiple factors such as substance abuse, poor diet, lack of exercise, inadequate seeking of physical care, side effects of medication, and health care factors [2]. In several countries, patients with SMI acknowledge practical and interpersonal barriers that interfere with their access to health care. Besides financial barriers, patients experience barriers such as long waiting times, crowded waiting rooms, and a hurried atmosphere. An interpersonal factor is the fear of patients that the emphasis of health care professionals is on their mental condition instead of their physical symptoms [3]. Health care professionals hinder the timely diagnosis and treatment of physical disease through poor attitude, inconsistent approaches, lack of awareness of physical diseases, insufficient skills and competencies, stigmatization, and lack of knowledge about the available services [3]. This leaves much room for improvement.

In The Netherlands, all citizens are compulsorily insured for health care [4,5] and registered at a GP practice. Outpatients with SMI are cared for by both mental health care services and a GP. A case manager performs the community care for chronic psychiatric patients. This mental health nurse is responsible for several patients with SMI and confers with a psychiatrist when necessary. The case manager has weekly to monthly contacts with his or her patients, and if necessary these contacts consist of home visits. The physical health care for patients with SMI is provided both by general practitioners (GPs) and mental health care services [2]. Additionally, families also form an important part of the support network for patients with SMI, performing informal care by ensuring medication compliance and providing support in recovery and risk management [6].

The World Psychiatric Association has made proposals for improvement of care [2], and health improvement programmes have been initiated [7,8]. However, patients and their family carers were not actively involved in the development of these proposals and improvement programmes. This is a pity, because they are potentially the most closely involved parties in a policy of shared decision making [6,9].

In order to improve the physical health care of patients with SMI, we wanted to discover what changes in the organization of this care are necessary. Therefore, we asked a convenience sample of both patients with SMI and their family carers to give their views on the current barriers and make suggestions on how to improve the logistics of the care.

## Methods

### Study design

This study was performed as a part of a larger study, investigating the preferences of the major stakeholders in the Dutch health care system for types of physical health care for patients with SMI. A qualitative study design was used to explore the experiences and ideas of patients with SMI and their family carers.

Patients with severe mental illness (SMI) were defined as individuals experiencing a reduction of general functioning due to their psychiatric disease, being in need of specialised care, and having received this care for at least two years [10-12]. Theoretically, all psychiatric diagnoses could present as SMI, but the major diagnosis groups of SMI are schizophrenia, bipolar disorder and personality disorder [12]. As patients with SMI are a hard-to-reach group, we pragmatically chose for the most feasible option. Staff members of the primary health centre where MO works asked patients with SMI whether they would like to share their own experiences in an interview with an independent investigator (FH). Also, in the mental health centre where FH works, mental health nurses from the community outreach team were requested to ask patients with SMI to join a small group interview. Information was given to the patients in face-to-face contacts and those who were interested received an information letter. Patients were eligible if they did not experience an exacerbation of their psychiatric symptoms and were able to express themselves.

Family carers of SMI patients were invited through Ypsilon, an organization for family members of patients with SMI. All participants filled out a form with information about age, sex and contact details. Additionally, patients were asked about the annual frequency of their visits to their GP and mental health care professional: psychiatrist as well as case manager. For the patients recruited through the primary health centre, the GP visiting rate is based on the file information. Family carers were asked about the degree of their relationship to the SMI patient and whether they were involved professionally in health care.

Both group and individual patient interviews were conducted by FH, while a professional facilitator mediated the group interview with family carers. A topic list was used for all interviews, consisting of the following items: experiences with physical health care, current barriers to physical health care and preventive care, responsibilities of professionals, and potential solutions. All interviews were taped, transcribed verbatim, and reviewed for accuracy by one investigator (FH).

### Ethical considerations

According to European Directive 2001/20/EC, this study is not an interventional trial but an exploratory inquiry concerning personal opinions on health care quality. Therefore, a medical ethics review is not needed.

Familiar staff members asked the stable patients if they would like to take part in interviews, conducted by an independent interviewer, about their experiences with physical health care. Explanation was given during face-to-face contact, and an information letter was provided. Patients were told that their participation was voluntary, and that participation or nonparticipation did not have any consequences for their treatment. Because patients and family carers volunteered to join and share their expertise, their participation with the interview after giving information about the research was regarded as consent.

### Data analysis

Two investigators (FH and MO) reviewed the transcripts and independently identified common topics. FH is a specialist trainee in psychiatry and PhD student, while MO is a general practitioner specialised in mental health in primary care. Discrepancies were resolved through discussion. For the purpose of checking back, a summary was sent to several participants of the group interviews, giving them the opportunity to agree or disagree with the content. During the individual interviews, the interviewer posed another question to check if her interpretation was correct. If this was deemed unclear when analysed, the data was not used. Thematic analysis was used for group responses into common topics. As analysis proceeded, increasingly in-depth coding categories were generated based on emerging thematic patterns. The topics were then grouped into themes. This process was supported by Kwalitan software [13].

## Results

Ten patients with SMI (4 men and 6 women) and 13 family carers (6 men and 7 women) were interviewed. The interviews were carried out as 7 individual interviews and 2 group interviews, of which one group consisted of 3 patients and the other of 14 family carers.

The mean age of the patients was 51 (41-66). All patients had a history of more than 2 years of treatment for their SMI by mental health care providers. Their diagnoses included schizophrenia, schizoaffective disorder, or bipolar disorder, and they were receiving care from both GP and mental health care providers, except for one patient, who was recently discharged from specialized care. The patients from the primary care centre visited the GP-practice, both for GP consultations and for consultations for blood pressure or diabetes controls with the practice nurse, annually ranging from 26 (n=1), 10-14 (n=2), 6-8 (n=4). The patients from the mental health centre consulted their GP rarely (n=2) or regularly (n=1) without being able to specify the exact number. All family carers were first-degree relatives of patients with SMI. Their mean age was 59 years (34 -73). Three of these family carers were professionally involved in physical health care: a nurse, an auxiliary nurse, and a medical doctor.

Three major themes emerged: needs of patients with SMI differ from the general population; professional roles and collaboration; and health monitoring and supporting a healthy lifestyle.

### Needs of patients with SMI

Patients and family carers emphasize that people with SMI differ from the general population and therefore it is necessary to tailor their health care to their specific needs. SMI patients experience a constant struggle to participate in society and cope with stress. Their level of participation in society and ability to ask for help varies with the severity of their psychiatric symptoms. Family carers are faced with the fact that patients living on their own are not fully capable of caring for themselves.

People, who have psychiatric problems, are easily disturbed by futile things. (Patient)

I’m afraid of doctor office visits; beforehand I worry about what will happen.

Therefore I’m very tired afterwards and that’s a pity. (Patient)

My son needed to visit his GP for stomach problems. Now, for the life of him he will not return, not to sit in a waiting room. (Family)

Patients often experience a barrier when making an appointment with their GP due to a sense of inferiority. Furthermore, most of them experience stress before and during the consultation, and while waiting for the results of laboratory assessments. Family carers add that some patients are not easily motivated to consult their GP. One father explained that the relation between a health care professional and a specific patient does not always work out well. He suggested that the professional should have an eye for problems within the relationship, and if this is the case, offer the opportunity for referral to another specialist.

One day they are motivated to visit their GP, but the next day they can just as well tell you that they don’t want to visit the GP. (Family)

If there is no match between the health care professional and the patient, the patient should be referred to another professional. Professionals should not be aggrieved by that. (Family)

### Professional collaboration and roles

The theme ‘professional roles and collaboration’ relates specifically to the roles of the GP and the mental health care provider and their collaboration.

#### Collaboration between health care professionals

I was using medication from my psychiatrist, but my GP was totally uninformed. (Patient)

My son is frightfully overweight and we can just wait for complications to happen. I don’t know who is responsible for his weight management. (Family)

Patients and family carers experience collaboration between GPs and mental health care professionals as either non-existent or sparse and non-systematic. This is perceived as an important barrier to receiving adequate health care. Furthermore, it is unclear for patients and family carers which professional is responsible for the diagnosis of physical co-morbidity, which can be caused by the psychotropic medication but is also present in the general population. Obesity, for instance, is a side effect of antipsychotic medication, and therefore the psychiatrist has the primary responsibility for the prevention and treatment of obesity. But in the general population, treatment of obesity is performed by the GPs. Patients and family carers suggest a systematic collaboration between GPs and mental health care providers as a solution to this problem. Family carers also propose an annual evaluation of the treatment plan with the patient, case manager, GP, and the family carer.

#### The role of the general practitioner

The GP is considered as the central professional who is contacted first about physical problems and should be informed about the current treatments by other specialists. Patients and family carers emphasize the importance of patients having confidence in their GP. This confidence builds slowly in patients with SMI and is easily damaged. As described above, making an appointment and consulting the GP can be stressful for such patients.

When an SMI patient calls for an appointment with the GP, the conversation starts with questions that make him nervous. To prevent stress these patients should be helped straight away. (Family)

Therefore, some patients and family carers advise that a marker should be put on the files of patients with SMI. This can be implemented as a pop-up in the computer system when opening a patient file to make the GP staff aware that this is not a regular patient, but a patient who requires a flexible approach and is often in need of reassurance.

Mark the file in order to emphasize that this is not a regular patient. If the patient calls and you are not aware of this, it can induce a lot of stress. (Family)

A marker has been added to my file and paramedical staff are aware now. It leads to an easier conversation. The person on the phone can reassure you. (Patient)

GPs should provide special attention to this group of patients and also pay attention to their mental health problems. Family carers desire that GPs pay unrequested house calls to patients with SMI to keep track of the current situation of the patient. The family practice nurse is another health professional who can play a role in supporting a healthy life style.

#### The role of the mental health care team

I’m appointed to another psychiatrist again. That’s unfortunate because I have to tell my story again. (Patient)

We discuss side effects on my initiative; they don’t ask for it. (Patient)

Patients often experience that their treatment is continued by a series of different psychiatrists. This means that they need to tell their life story again to each new psychiatrist. Besides, physical symptoms are not discussed in the consultation with the psychiatrist, and not all mental health care staff ask patients about the side effects of medication. Patients and family carers would like to receive more information about their medication and potential side effects. They find that the prescriber, often the psychiatrist, is responsible for providing them with this information and for notifying the GP. Family carers often signal that mental health care staff do not inform them about the risks of physical disorders, and they emphasize that this should be improved.

Sometimes he is so absorbed in his compulsions that he finds no time for cooking or eating. Somebody has to take notice of that and take action (Family)

Some patients need the support of their case manager in order to adequately seek treatment from their GP. This support should be individualized according to the specific needs of each patient.

### Monitoring and supporting a healthy lifestyle

When a laboratory assessment needs to be performed, patients prefer blood samples to be taken at their GP practice close to their home, and they request that the results are discussed as soon as possible in order to reduce the stress and fretting about the possible results.

I would like to know the results as soon as possible. So you don’t have to brood for two weeks if everything will be all right. (Patient)

Patients explain that a laboratory assessment is sometimes performed by the GP only weeks after the psychiatrist has performed it, or vice versa. They are positive about their physical health being closely monitored, but they find it a burden that assessments are repeated unnecessarily due to a lack of collaboration amongst professionals.

Family carers expressed the view that there are too few activities for patients with SMI, and that the consequent boredom leads to physical disorders. They advise an increase in the number of these activities. Most patients like joining regular sports facilities or clubs when possible. Patients experience that when they are in a phase of isolation due to their psychiatric symptoms, doing sport with other patients can be a safe alternative to a regular sports facility. Patients emphasize that the ultimate goal should be to join a regular facility. Not all patients are motivated to do exercise, even though they are aware of its benefits. Similar responses were given in relation to the cessation of smoking. Smoking is seen as a comforting activity.

Giving up smoking is a difficult question. In some way or other, I don’t want to quit.

I’m not up to it. Smoking is so tasty. (Patient)

Family and patients also mentioned dental care as an important aspect of healthy living. Furthermore, the pharmacist is an important possible source of information for pharmacotherapy.

## Discussion

### Main results

Both patients and family carers are aware of the general paradox that patients with SMI require a strong support system in order to participate in society in the same way as the general population. This phenomenon means that patients with SMI need a supportive approach by health care professionals in order to obtain equal health care. This supportive approach should take into account the specific vulnerabilities of patients with SMI, including the difficulty they find in building up a therapeutic relationship with professionals and the fact that they are easily stressed. These vulnerabilities may change over time and differ with the severity of psychiatric symptoms.

Patients and family carers point out the problem of barriers to well-organized physical healthcare but are not advocating radical changes in the organizational structure. Patients and family carers urge the organization of systematic and structural collaboration between GP and mental health care services, with clearly defined responsibilities. Also, professionals need to apply a more tailored approach when caring for patients with SMI. For general practice this means keeping a close eye on these patients, both when requested and when unrequested. For mental health care, a signalling role for physical problems and risks is required as well as support and training for the patient in consulting the GP. Having knowledge of specific health risks is a prerequisite for case managers to be able to perform these tasks.

The improvement of physical health, including life style guidance, is appreciated as an important part of health care. There is no clear preference as to which professional should provide this care. The information on drug treatment and the side effects of psychiatric medication should be given by the psychiatrist.

Qualitative research methods are used to study specific stakeholders in specific situations. However, based on the publication of the World Psychiatric Association about barriers to care [2], we expect that our findings can be generalized to other countries with similar health care systems with separated primary care and mental health care. We expect that the need to create a logistical system that acknowledges the specific needs of these patients will not be limited to the Dutch health care system.

### Strength and limitations

This is the first study exploring the vision of both patients and family carers on the organization of health care. Patients and families felt free to discuss their opinions in the interviews. Patients were motivated to join the study, and they were able to reflect on the organization of health care. It should be noted that all patients were in a stable situation and their psychiatric symptoms were reasonably well controlled. This means that the information on the needs of patients in crisis is retrospective. Patients came from different mental health care providers and had different GPs. Since this was a convenience sample, selection bias might be present with an overrepresentation of people with a specific interest in this subject. Also, verbally stronger patients may be overrepresented due to the participation based on invitation. However, family carers also represent patients who are less verbally strong and even more incapable in requesting care.

Family carers noted barriers other than those noted by patients, and therefore they gave additional information; they were a relevant source of extra information. The family carers were recruited from one region of The Netherlands, as this is part of the organizational structure of Ypsilon. They had experience with different mental health care providers and GPs. We expect no specific regional aspects that limit the generalization of the findings of this study to the rest of the Netherlands.

### Comparison with literature

In a review of qualitative studies, Chadwick et al. [3] describe how patients experience barriers to care due to long waiting times, crowded waiting rooms and a hurried atmosphere, in addition to financial and logistical aspects. In our study, financial constraints were not mentioned as a barrier to care, but we did find that SMI patients are easily stressed. Chadwick et al. [3] find that isolation is a risk, resulting in fewer medical visits [2]. This matches our finding that extra support is necessary when patients are in a phase of severe psychiatric symptoms and tend to isolate themselves.

In apparent contradiction to our finding that SMI patients experience a barrier to making appointments at the GP practice, the mean frequency of their contact with the GP is higher than that of members of the general population with a chronic disease [14]. The general population has a mean of 3.8 consultations annually with the GP, including phone contact [15]. This higher contact frequency is interpreted by the authors [14] as a consequence of their heterogeneous comorbidity. Another explanation of these superficially inconsistent findings may be the effectiveness of their GP visits. Patients with SMI find it difficult to express their symptoms and often interpret internal stimuli in a different way to the general population [2]. As a consequence, they have to make extra appointments, which might give them stress. Furthermore, it should be noted that there is a subgroup of patients, which seldom visits the GP even though they experience physical health problems [16].

In other qualitative studies, barriers to optimal health were also the result of poor coordination and the lack of knowledge of mental health care staff in signalling physical health problems [3]. Poor collaboration between health care specialists is also identified as a barrier in a literature review conducted by experts on physical health in patients with SMI [2]. Therefore, lack of collaboration is not specifically a Dutch problem but is present in several countries where a separation between primary care and mental health care exists. Most mental health nurses describe identifying physical symptoms as part of their job. However, in line with our findings, they emphasize the need for more education on physical health risks in order to perform this job properly. This need for education is most apparent if nurses do not have a general nursing background [17].

Patients and family carers request that SMI patients are treated differently to ensure that they have equal opportunities to acquiring optimal health care. A similar request was formulated as a request for parity in health care by a meeting of expert professionals on physical health care for patients with SMI [18]. This represents more than a simple wish or request; it is a basic human right of patients with SMI to receive this care, like all patients with a higher burden of physical illness [19]. Although the labelling of files can be seen as stigmatising, it is meant as a way of promoting participation and therefore reducing stigma. Labelling or flagging a file should be used as a system to draw attention to a group of patients with specific susceptibilities and risks. The labelling of a file and the consequent difference in treatment can be compared with the provision of a wheelchair to a paralysis patient: it should be seen as a prosthesis for participation in society rather than stigma and exclusion. There is a related need to train staff in the interpersonal skills needed for such labelled patients.

## Conclusions

Patients and family carers state that an optimal approach for caring for the physical health of patients with SMI requires a different professional approach than the routine care for the general public. This approach can and should be accomplished within the usual organizational structure, but professionals should tailor the care provided to the needs of patients with SMI.

Collaboration between mental health care providers and GPs is very important, especially at a time when most patients with SMI are treated as outpatients. This requires an investment in professional time for inter-professional consultations. Both patients and family carers emphasize the urgent need for collaboration between mental health care professionals and GPs. The care responsibilities need to be clearly agreed between these professionals, and education should take place concerning specific health risks. Mental health care professionals should support a patient in obtaining necessary health care from the GP. Additionally, GPs should make their care more easily accessible by putting a label on the files of patients with SMI in order to reduce the stress of patients when making an appointment. Furthermore, laboratory assessments should be performed close to the patient’s home, and the results of these tests and the consequences should be discussed with the patient as soon as possible. These changes should be implemented into the current care system in order to ensure that SMI patients receive the health care they need.

## Abbreviations

SMI: Severe mental illness; GP: General practitioner.

## Competing interests

The authors declare that they have no competing interests.

## Authors’ contributions

FvH has made substantial contributions to the conception and design of the study, performed some of the interviews, carried out the qualitative analysis and has drafted the manuscript. MO has made substantial contributions to the design of the study, carried out the qualitative analysis and has revised the manuscript for intellectual content. AL has made substantial contributions to the conception and design of the study and has critically revised the manuscript for intellectual content. All authors read and approved the final manuscript.

## Pre-publication history

The pre-publication history for this paper can be accessed here:

http://www.biomedcentral.com/1472-6963/13/426/prepub
